# Banker Plant Efficacy to Boost Natural Predators for Management of Field Populations of *Scirtothrips dorsalis* Hood (Thysanoptera Thripidae) in Strawberries

**DOI:** 10.3390/insects15100776

**Published:** 2024-10-08

**Authors:** Allan Busuulwa, Alexandra M. Revynthi, Oscar E. Liburd, Sriyanka Lahiri

**Affiliations:** 1 Gulf Coast Research and Education Center, Entomology and Nematology Department, University of Florida, Wimauma, FL 33598, USA; 2 Tropical Research and Education Center, Entomology and Nematology Department, University of Florida, Homestead, FL 33031, USA; 3 Entomology and Nematology Department, University of Florida, Gainesville, FL 32608, USA

**Keywords:** *Fragaria x ananassa*, flowering plants, thrips, integrated pest management, biological control

## Abstract

**Simple Summary:**

Several thrips species (Hymenoptera: Thripidae) are important agricultural pests of many crops around the world. In Florida, the invasive chilli thrips, *Scirtothrips dorsalis* Hood, originating from Southeast Asia, is an important pest of blueberries and strawberries. Growers around the state rely mainly on insecticides to manage *S. dorsalis* populations; however, *S. dorsalis* is currently showing reduced susceptibility to selected insecticides. To expand the management options for *S. dorsalis*, we investigated the efficacy of utilizing flowering plants (banker plants) to attract naturally occurring biological control agents (predators) in a strawberry field to suppress *S. dorsalis* populations. Among the tested banker plants, ornamental pepper and sweet alyssum emerged as promising candidates given their ability to continuously produce flowers that attracted thrips predators throughout the growing season. Overall, we identified two potential thrips predators (*Geocoris* spp. and *Orius* spp.) that were attracted by the banker plants; however, their numbers were too low to effectively suppress *S. dorsalis* populations. These results showed that sweet alyssum and ornamental pepper may serve as insectary plants to support the establishment of *Orius* and *Geocoris* species in the field. Further research should focus on the release of commercially available *Orius* species on the banker plants early in the season to facilitate population buildup of the predator and enable the suppression of *S. dorsalis* on strawberry plants.

**Abstract:**

Since 2015, *Scirtothrips dorsalis* Hood has emerged as the main pest of strawberries in Florida. Given the limited management options, there is a recognized need to expand on the management strategies for this pest. Therefore, we explored the possibility of using banker plants to recruit naturally occurring predators of thrips into strawberry fields to suppress *S. dorsalis*. The study began in the 2021–2022 strawberry season where five banker plants were screened to determine which ones could consistently attract thrips predators by flowering throughout the strawberry season. *Capsicum annum* L. (ornamental pepper) and *Lobularia maritima* L. (sweet alyssum) were selected for further evaluation. In the 2022–2023 strawberry season, using a randomized complete block design we assessed the capability of these banker plants to attract thrips predators into the strawberry field. In addition, we examined how the banker plant distance from the strawberry plants influenced the *S. dorsalis* pest suppression. Our results showed that strawberries located within 3.7 m of ornamental pepper plants had less leaf damage from *S. dorsalis* compared with those farther away, which may result from the repellent effect of the ornamental peppers. Additionally, *Geocoris* spp. and *Orius* spp. were identified as the main thrips predators in the system, although in relatively low numbers. Therefore, these results highlight the potential of incorporating ornamental pepper as a banker plant in strawberry production. Additional applications of this research are explored below.

## 1. Introduction

Florida is the second largest producer of strawberries (*Fragaria x ananassa* Duchesne (Rosaceae)) and the largest producer of winter strawberries in the United States [1,2]. In the 2022–2023 strawberry season, Florida’s strawberry production generated over half a billion dollars in revenue (USDA/NASS-2023). However, strawberry producers face many biotic challenges, especially from arthropod pests and diseases. Strawberries in Florida are commonly infested by several thrips (Thysanoptera: Thripidae), particularly *Frankliniella* species such as *Frankliniella occidentalis* Pergande (western flower thrips), *Frankliniella schultzei* Trybom (blossom thrips), *Frankliniella bispinosa* Morgan (Florida flower thrips) [3,4], and the invasive and highly polyphagous *Scirtothrips dorsalis* Hood, commonly known as chilli thrips [5]. Since 2015, *S. dorsalis* has emerged as the most severe pest of strawberries in the state [5,6,7] and has been reported to infest other important crops such as roses (*Rosa* X “Radrazz”, Rosaceae) [8], peppers (*Capsicum* spp., Solanaceae) [9], and blueberries (*Ericaceae* spp., Ericaceae) [10].

The life cycle of *S. dorsalis* consists of six distinct stages: eggs; two actively feeding larval stages; two inactive, non-feeding stages, namely, the prepupa and pupa; and a feeding adult stage. With the exception of the eggs that are oviposited inside the leaf tissue, all life stages of *S. dorsalis* typically conceal themselves behind the fruit calyx of the host plant where they complete their life cycle [11]. A single *S. dorsalis* female can lay two to four eggs per day on a chilli plant, with a total of 60 to 200 eggs laid throughout her lifetime [12]. The developmental time of *S. dorsalis* from egg to adult is significantly influenced by environmental conditions, particularly temperature [13]. Average temperatures between 24 °C and 28 °C have been reported to promote rapid growth, with an average development time of 12 to 15 days [14]. Given that the average temperature particularly in Plant City where the majority of strawberry growing in Florida takes place ranges between 12 °C to 28 °C (U.S Climate Data 2024) during the strawberry season (October to March), *S. dorsalis* can rapidly complete multiple generations, resulting in season-long infestation. In strawberries, the adult and larval stages of *S. dorsalis* damage the plant by feeding on both the fruit and the young causing leaf bronzing along the veins and petioles with subsequent darkening, curling and hardening, and fruit bronzing [6].

The management of *S. dorsalis* in strawberries is centered around the use of reduced risk insecticides [7,15,16] and the release of predatory mites, particularly *Neoseiulus cucumeris* Oudemans, and *Amblyseius swirskii* (Athias-Henriot) (Mesostigmata: Phytoseiidae) [5,17,18,19]. However, continued reliance on insecticides for *S. dorsalis* management has resulted in reduced susceptibility to these compounds [20,21], threatening the loss of one of the few existing *S. dorsalis* management strategies in strawberries. Therefore, the need for the development of new innovative management strategies to maintain effective control of *S. dorsalis* is warranted. To expand the management options for *S. dorsalis*, we investigated whether banker plants could be used to attract beneficial insects, specifically natural predators found near strawberry fields, to suppress *S. dorsalis*.

Banker plants are plants grown either near or within agricultural fields with the goal of supporting and enhancing the establishment and survival of beneficial organisms, particularly naturally occurring predators and parasitoids [22]. These plants offer crucial food resources, such as alternative prey, pollen, nectar, or plant fluids, which help to sustain predator populations, particularly when pest numbers are low, enabling the predators to persist and to rapidly respond to pest outbreaks [23,24,25].

In addition to providing essential food resources, multiple studies have attributed the success of banker plants in controlling pest species to their ability to offer natural predators shelter from environmental stress, as well as ideal reproductive substrates for oviposition and development [24,25,26,27].

For example, intercropping sweet alyssum, *Lobularia maritima* (L.) Desv. (Brassicales: Brassicaceae) with squash *Cucurbita pepo* L. (Cucurbitales: Cucurbitaceae) was shown to enhance the abundance and diversity of natural enemies of the whitefly *Bemisia tabaci* Gennadius (Hemiptera: Aleyrodidae) [28]. The common marigold *Calendula officinalis* L. (Asterales: Asteraceae), yarrow, *Achillea millefolium* L. (Asterales: Asteraceae), and sweet alyssum have been reported to enhance biological control of the oleander aphid *Aphis nerii* Boyer de Fonscolombe (Hemiptera: Aphididae) on ornamental oleanders, *Nerium oleander* L. (Gentianales: Apocynaceae) [26]. Cowpea, *Vigna unguiculata* (L.) Walp. (Fabales: Fabaceae), has been reported to attract beneficial arthropods such as hoverflies (Diptera: Syrphidae), lady beetles (Coleoptera: Coccinellidae), and a few species of parasitoids (Hymenoptera: Ichneumonidae) [25] that could suppress various pests such as aphids and whiteflies. Field studies in vegetable crops such as tomatoes (*Solanum lycopersicon* L., Solanales: Solanaceae), eggplants (*Solanum melongena* L., Solanales: Solanaceae), and onions (*Allium cepa* L., Asparagales: Amaryllidaceae) demonstrated that using marigold as a banker plant improved insect pest management by increasing the density and diversity of natural enemies present in the fields [29,30]. Using banker plants as insectary plants for biological control of various insect pests, particularly in vegetables, has therefore produced promising results [25].

In strawberry production, the potential of using banker plants to suppress pests has been demonstrated primarily in greenhouses [31]. Kordestani et al. (2020) [32] showed that *Orius laevigatus* Say (Hemiptera: Anthocoridae), when tested in a cropping system composed of marigolds, strawberries, and green beans (*Phaseolus vulgaris* L., Fabales: Fabaceae), preferred laying eggs on marigolds over green beans, which led to higher suppression of western flower thrips on strawberries in the greenhouse. When used in combination with biological control agents, banker plants can lead to the season-long suppression of various pests without the need of conducting additional releases of biological control agents [26,28,33].

Although banker plants provide many ecosystem services, their adoption in many cropping systems remains limited due to concerns about efficacy, reliability, and cost. Even for common pests like *Aphis gossypii* Glover (Hemiptera: Aphididae), there is no consensus on the best banker plant system, as there is a lack of recommendations for effective plant–host–natural enemy combinations that ensure reliable control for growers [22]. Additionally, in field conditions, a single crop can host multiple pests, creating a complex system influenced by various factors affecting species interactions, making it hard to recommend a single banker plant to address all pest issues. Furthermore, the development and implementation of banker plant systems has also been hindered by limited research and, in some cases, a lack of experimental rigor [23].

As previously mentioned, most research on banker plants in strawberry systems has been conducted in controlled greenhouse settings, focusing on a single pest species. The success of banker plants in these environments highlights the need to investigate their effectiveness in field conditions. This exploration is essential to determine if a specific banker plant can increase the abundance or diversity of natural predators and whether this leads to improved suppression of *S. dorsalis* in strawberry crops. Therefore, this study had two main objectives. The first was to identify potential banker plants that could continuously produce flowers throughout the strawberry growing season. The second was to evaluate the effectiveness of these plants in attracting natural predators of *S. dorsalis* and to determine whether these predators could help suppress *S. dorsalis* populations in strawberry fields. The rationale for focusing on banker plants capable of flowering throughout the season was to determine whether these plants could consistently attract naturally occurring *S. dorsalis* predators by continuously providing nectar and pollen. This information is crucial for selecting suitable banker plants that can enhance the abundance and/or diversity of naturally occurring predators. Additionally, the findings from this study provide valuable insights into whether the establishment of banker plants near strawberry crops could eliminate the need for releasing augmentative biological control agents or relying on insecticides for *S. dorsalis* management in strawberries.

We hypothesized that banker plants that continuously produce flowers throughout the strawberry growing season would attract natural predators of thrips, leading to suppression of *S. dorsalis* populations in strawberry fields.

## 2. Materials and Methods

### 2.1. 2021–2022 Banker Plant Screening Study

In 2021, five potential banker plants were identified from the published literature and screened in order to identify those capable of flowering throughout the strawberry growing season and determine their ability to attract thrips predators, specifically the minute pirate bug *Orius* spp. (Hemiptera: Anthocoridae) [34], the big-eyed bug (Hemiptera: Geocoridae) [35], and predatory thrips *Franklinothrips vespiformis* Crawford (Thysanoptera: Thripidae) [36]. The banker plants that were selected for testing included cowpea, sweet alyssum, buckwheat *Fagopyrum esculentum* Moench (Caryophyllales: Polygonaceae), ornamental pepper *Capsicum annum* L. (Solanales: Solanaceae), and sunn hemp *Crotalaria juncea* L. (Fabales: Fabaceae). All banker plant seeds were purchases from Mountain Valley Seed, Salt Lake City, UT, USA.

All experiments were carried out at the University of Florida Gulf Coast Research and Education Center (GCREC), Wimauma, Florida (latitude, 27.7656; longitude, −82.2283). The screening experiment began in August 2021 with the preparation of raised beds on which banker plants and strawberries were grown. Strawberry is grown as an annual crop in Florida (between September to April) on raised beds. Raised beds are used to create a well-drained soil environment to ensure that plant roots receive adequate oxygen for survival during heavy rainfall. The beds were approximately 20 cm high and covered with black, virtually impermeable plastic mulch (Blockade, Berry Plastics, Sarasota, FL, USA). Before laying the black mulch, drip lines were installed within the beds to establish a drip irrigation system for watering and fertigating the plants. Black plastic mulch was used because it provides excellent weed control, aids moisture retention, and provides insulation to keep the roots warm during cold periods [37]. The beds were fumigated with a soil fungicide/nematicide 1,3-dichloropropene + chloropicrin (Telone C-35, 280 L ha^−1^, Telon Ag Solutions, Pinehurst, NC, USA) and fertilized with 2.2 kg/ha of nitrogen. Additionally, at the beginning of the field season, flumioxazin (Chateau^®^ Herbicide SW, 0.44 L per hectare, Valent^®^) was applied once around the strawberry bed borders for weed management.

**Figure 1:**
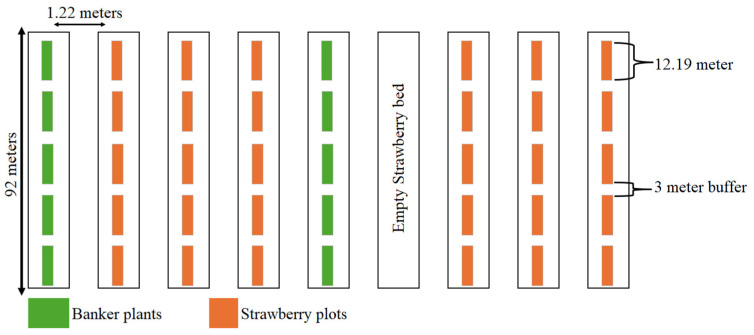
Field experimental design of the 2021–2022 banker plant screening study.

Eight raised beds, spaced 1.22 m apart (center-to-center), were used for this field study. Strawberry plants were planted in six of these raised beds, while the banker plants were planted in the remaining two (Figure 1). Using a completely randomized experimental design, banker plants were placed side by side to form 12.19 m long strips, with each strip consisting of five banker plants. These strips were replicated five times on each raised bed containing banker plants, with each replication separated by a 3 m buffer. Strawberry plants on beds were planted in plots that were parallel with the banker plant strips, maintaining the same spacing between the replicates as in the banker plants.

Banker plants were hand planted directly as seeds (planting date 7 October 2021), without the application of any chemical treatments, at a depth of 5.08 cm. A spacing of 3 cm was maintained between each plant, all situated on raised beds that were supplied with a consistent drip irrigation system. Strawberry plots containing 12–15 strawberry (“Florida Brilliance variety”) transplants procured from a nursery (GW Allen, Centreville, NJ, Canada) were planted in plots measuring 12.19 m by 1.22 m at a depth of 15 cm. The distance of the successive strawberry beds from the banker plant beds were 1.2, 2.4, 3.7, and 5.0 m. Strawberry transplants were planted in early October, in the same week in which hand seeding of the banker plants occurred. All plants were maintained following local extension protocols with regards to plant disease management; however, no insecticide applications were conducted except treatment with *Bacillus thuringiensis*, subsp. *kurstaki*, strain ABTS-351 (DiPel DF, Valent USA LLC, San Ramon, CA, USA), to manage lepidopteran pests at the beginning of the season in October.

Strawberry plants were sampled once every 14 days for *S. dorsalis*. At each sampling point (strawberry plot), five randomly selected young leaves and flowers were collected in separate 3.7 L sealed plastic storage bags (Ziplock, SC Johnson & Sons Inc., Racine, WI, USA) and kept in the freezer until further processing in the laboratory. Additionally, five random plants at each sampling point were visually rated for 2 min to assess the leaf damage index during each sampling event. The leaf damage index used to assess *S. dorsalis* foliar damage on strawberries followed a similar approach to that described by Lahiri and Yambisa [15,18]. In this system, a score of zero indicated no damage, while a score of 1 represented less than 19% bronzing and reddening of leaf veins and petioles. A score of 2 corresponded to 20–39% damage, a score of 3 to 40–59% damage, and a score of 4 to 60–79% damage. Damage exceeding 80% was assigned the highest score of 5.

Every 7 days, all strawberry fruits from each plot were harvested and graded. The fruits were graded on marketable standards, using criterion such as firmness; uniform ripeness; and the absence of mold, mechanical defects, and insect damage [38]. After grading, the marketable and non-marketable fruits from each plot were counted and weighed. In the laboratory, leaves and strawberry flowers were vigorously stirred in a plastic bag containing 70% ethanol (Thermo Fisher Scientific, Hampton, NH, USA) to dislodge all thrips life stages present on the plant tissue [18,39]. Thrips in alcohol were then placed under a microscope for species identification. The number of *S*. *dorsalis* larvae and adults were counted and recorded. For trapping and assessing natural enemies, yellow sticky cards were randomly placed in both banker plant and strawberry plots and replaced every 14 days. Sticky cards collected from the field were individually placed in a sealed plastic bag and stored in the freezer until processing. Natural enemies trapped on the sticky cards were counted and identified under a stereomicroscope in the laboratory. The collection and assessment of natural enemies was conducted during the entire strawberry field season from November 2021 through March 2022. Banker plants that senesced early during this study were eliminated from further field evaluation in the following year.

### 2.2. 2022–2023 Banker Plant Evaluation Study

**Figure 2:**
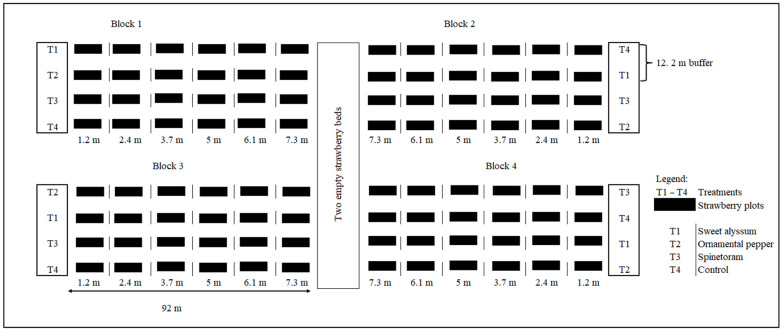
Field experimental design of the 2022–2023 banker plant evaluation study.

Following the 2021–2022 banker plant screening study, sweet alyssum and ornamental pepper were identified as ideal candidates for further field evaluation. This was because both plants consistently produced flowers throughout the strawberry season, while all the other banker plants senesced early during the field season. In the 2022–2023 study, a randomized complete block design with four treatments (Figure 2) was used to evaluate the potential of ornamental pepper and sweet alyssum in attracting natural thrips predators for *S. dorsalis* suppression. In addition to the two banker plants, an insecticide treatment spinetoram (Radiant^®^ SC, Corteva Agriscience, Indianapolis, IN, USA) was introduced into the study to compare the effectiveness of natural enemies in suppressing *S. dorsalis* with the industry standard chemical control tool. Spinetoram was applied twice during the season, in November and January, following the manufacturer’s recommended rate for strawberries of 0.88 L/ha. Spinetoram was first applied in November when the initial signs of *S. dorsalis* foliar damage appeared on the strawberry plants and again in January following a sharp increase in the pest population. In both instances, the application was performed ensuring thorough plant coverage until the plants were completely wet.

Strawberry and banker plant beds were established following the same protocol as described in the 2021–2022 study. Control strawberry plots without any banker plants or spinetoram treatment were included to serve as a baseline for comparison. Each experimental plot measured 3 m × 1.2 m, with banker plant treatments separated by a 12.2 m buffer. To provide a deeper understanding of the effect of banker plants, strawberry plots were established at distances of 1.2 m, 2.44 m, 3.7 m, 5 m, 6 m, and 7.3 m away from the banker plants. Each treatment included four replicates, with strawberry beds positioned at the specified distances (Figure 2).

Strawberry transplants were planted in mid-October in 2022 at a spacing of 0.1 m; a total of 20 plants were planted in each plot. Ornamental pepper plants (Mountain Valley Seed, UT, USA) were initially planted in the greenhouse (28 ± 1 °C, 40 ± 5% RH, and 16:8 h L:D) to ensure timely flowering in the field. Upon reaching full maturity, the pepper plants were transplanted into the field at a depth of 0.1 m and a spacing of 0.2 m, 10 days before the strawberries were planted. This approach was adopted to ensure proper ornamental pepper transplant establishment and to minimize damage from the 14-day overhead irrigation required for the strawberry transplants. Sweet alyssum seeds were sown directly into the soil at a rate of 3 g per plot and a depth of 0.04 m. All plots were managed following the strawberry growing extension protocols as described for the 2021–2022 field season.

Field sampling was performed following the sampling protocol of the 2021–2022 field study. The only modifications made were sampling eight strawberry plants per plot instead of five and weighing only the marketable strawberry fruits from each plot after grading. For trapping and assessing natural enemies, yellow sticky cards were placed in each banker plant plot and in three random strawberry plots in each treatment. The sticky cards were collected and replaced every 14 days. The sticky cards collected from the field were labeled according to treatments, individually placed in a sealed plastic Ziploc^®^ bag (Johnson & Son, Inc., Racine, WI, USA), and stored in the freezer until processing. During processing, natural enemies trapped on the sticky cards were identified and counted under a stereomicroscope in the laboratory. The collection and assessment of natural enemies were conducted during the entire strawberry field season.

### 2.3. Statistical Analysis

The data analysis was conducted using Generalized Linear Mixed Effects Models (GLMM) in R, version 4.3.0 [40]. Model fitting was performed using the glmmTMB package [41]. In the models, the response variables included the number of *S. dorsalis* collected from the strawberry plant leaves, the marketable yield (measured by both weight and total fruit count), and the damage index of the strawberry leaves. The number of *S. dorsalis*, the number of predators caught on the sticky cards, and the number of marketable fruits were modeled using a negative binomial distribution, while strawberry leaf damage was modeled using a beta binomial distribution. This was followed by an analysis of deviance (Chi-square test (χ^2^)), performed using the ANOVA function from the car package [42]. Additionally, when a significant interaction term was detected, we performed linear contrasts using the Tukey adjustment for multiple comparisons with the “emmeans” function from the “emmeans” package [43].

## 3. Results

### 3.1. 2021–2022 Study

**Figure 3:**
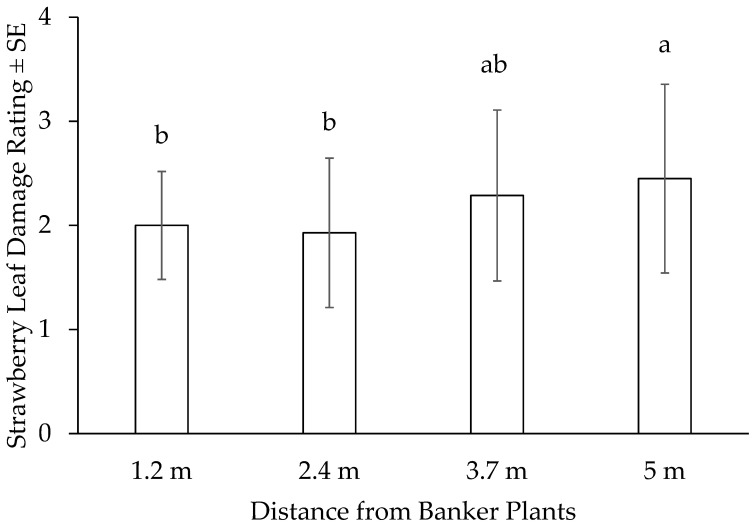
The damage ratings of plants located closer to or farther away from the banker plants. Damage ratings that differ significantly based on linear contrasts (Tukey *p* < 0.05) are indicated by different letters on the bars. A score of 1 represents less than 10% bronzing and reddening of leaf veins and petioles, a score of 2 corresponds to 20–39% damage, a score of 3 to 40–50% damage, and a score of 4 to 60–70% damage. Damage exceeding 80% is assigned the highest score of 5.

The analysis of deviance results indicated that the presence of banker plant strips did not have a significant impact (χ^2^ = 8.5175, df = 3, *p* = 0.480) on the number of *S. dorsalis* found on the strawberry leaves; however, the distance from the banker plants had a significant effect (χ^2^ = 11.449, df = 3, *p* = 0.001) on the leaf damage index of the strawberry leaves. Strawberry plots that were at 5 m from the banker plants had a higher damage rating compared with those that were closer to the banker plants (Figure 3).

**Figure 4:**
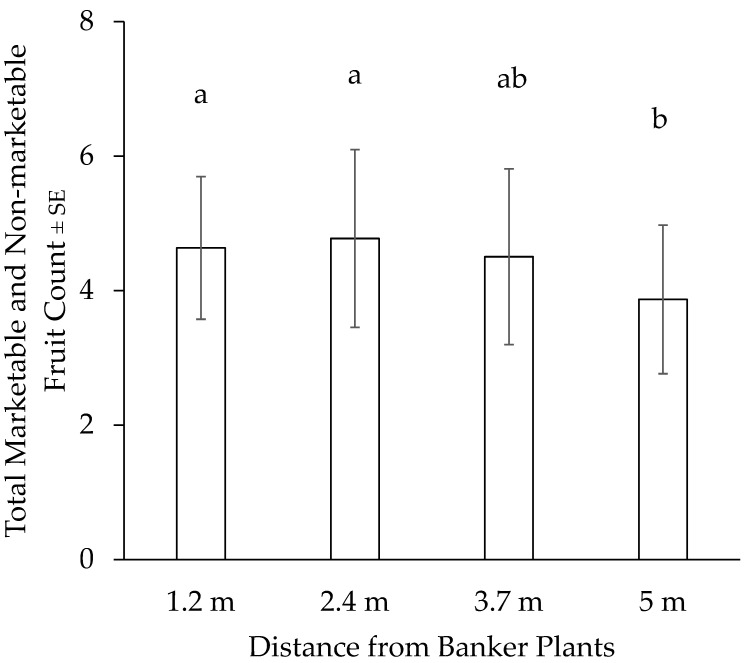
Total fruit count of strawberry plants in plots closer to and farther away from banker plant strips. Fruit counts that differ based on linear contrasts (Tukey *p* < 0.05) are differentiated by different letters on bars.

There were no significant differences in the number of strawberry fruits (χ^2^ = 4.242, df = 3, *p* = 0.2365) between strawberry plants in plots that were close to the banker plants compared with those that were farther away from the banker plants. However, strawberry plants that were closer to the banker plants had higher overall fruit counts (both marketable and non-marketable) (Figure 4) compared with those that were farther away (χ^2^ = 9.0531, df = 3, *p* = 0.02859).

**Figure 5:**
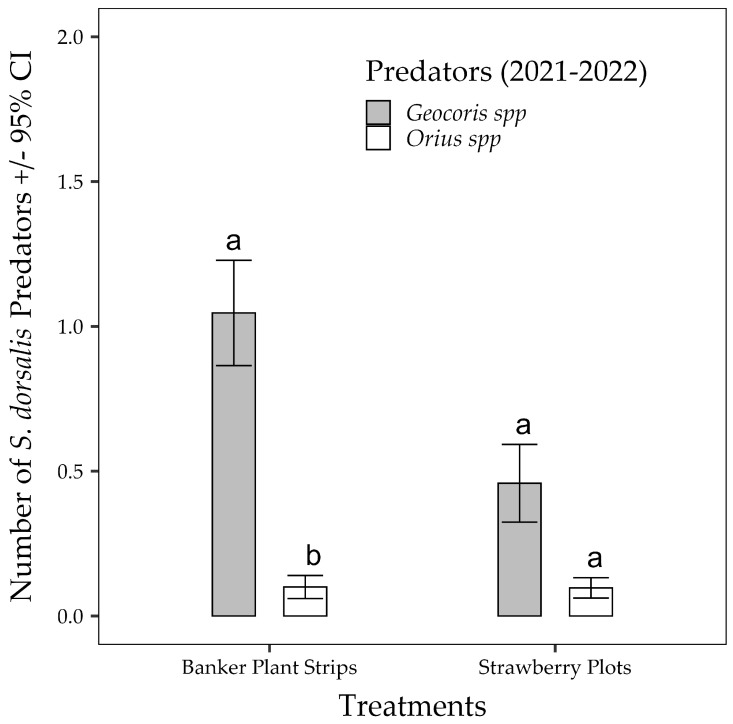
The number of *S. dorsalis* predators captured on yellow sticky cards placed in the banker plants and strawberry plots during the 2021–2022 strawberry season. Comparisons were made within each treatment. The number of predators showing significant differences based on linear contrasts (Tukey *p* < 0.05) are indicated by different letters on the bars.

Throughout the season, the number of thrips predators caught on the sticky cards was very low. There was a significant difference (χ^2^ = 4.3258, df = 1, *p* = 0.038) in the numbers of thrips predators captured in traps within the banker plant strips compared with those in the strawberry plots. The two main thrips predators captured were *Geocoris* spp. and *Orius* spp. (Figure 5). Among these, *Geocoris* spp. was the predominant predator, with a higher average number of the predator occurring in the banker plant strips (0.10 confidence interval [CI]: 0.82–1.30) compared with *Orius* spp. (0.01 CI: 0.012–0.32), while the predators occurred in similar proportions in the strawberry plots.

### 3.2. 2022–2023 Study

**Figure 6:**
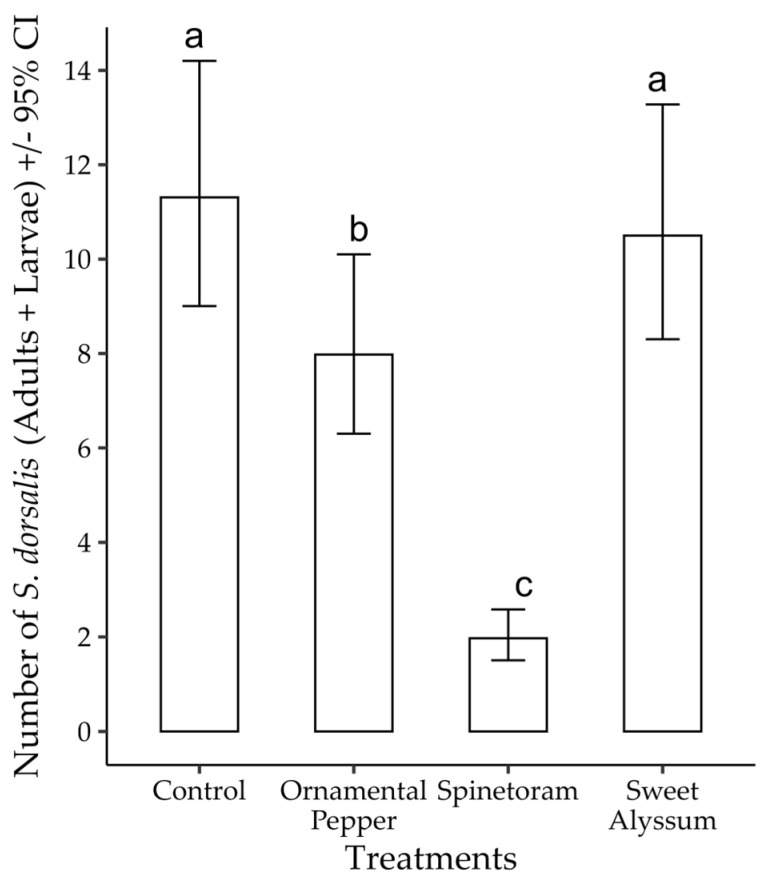
Number of *S. dorsalis* found on strawberry leaves in plots adjacent to all treatments in the 2022–2023 strawberry season. Contrasts that differ significantly (Tukey *p* < 0.05) are indicated by different letters on bars.

The analysis of deviance indicated that the grown banker plants (treatments) had a significant impact on the population of *S. dorsalis* found in the strawberry plots (χ^2^ = 243.024, df = 3, *p* < 0.001). However, the interaction term between banker plant treatments and the planting distance had no effect on the number of *S. dorsalis* (χ^2^ = 18.658, df = 15, *p* = 0.23) found in strawberry plots adjacent to the treatment. Within the treatments, the spinetoram treatment (1.97, CI: 1.51–2.58) had the average lowest number of *S. dorsalis* in all plots compared with the control (11.31, CI 9.00–14.20) and sweet alyssum (10.50, CI: 8.30–13.27), which had the highest number of *S. dorsalis* (Figure 6).

**Figure 7:**
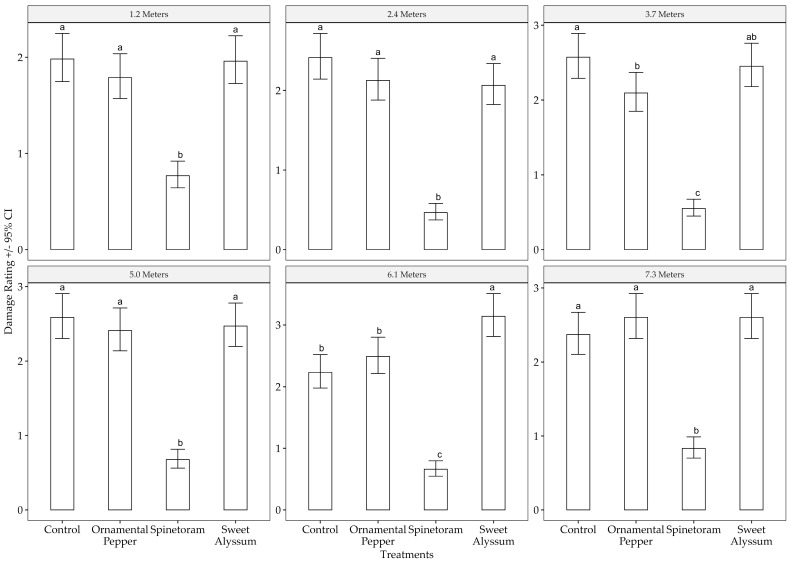
Strawberry leaf damage ratings among treatments at each distance from the banker plants. Bars with different letters indicate significant differences (Tukey *p* < 0.05) within each panel. A score of 1 indicates less than 10% bronzing and reddening of veins and petioles, 2 corresponds to 20–39% damage, 3 to 40–50%, 4 to 60–70%, and 5 represents over 80% damage.

Both banker plants and spinetoram treatments significantly reduced the strawberry leaf damage index (χ^2^ = 1277.696, df = 3, *p* < 0.001). Plants closer to the banker plants had less damage than those farther away (χ^2^ = 24.765, df = 5, *p* < 0.001), and this effect varied by treatment (χ^2^ = 96.121, df = 15, *p* < 0.001). Spinetoram treatment consistently had the lowest leaf damage index (Figure 7) compared with all other treatments at all distances. In contrast, the control treatment and the sweet alyssum treatment had the highest damage indices across all distances, with the highest damage observed when plants were 7.3 m away from the banker plants (Figure 7).

**Figure 8:**
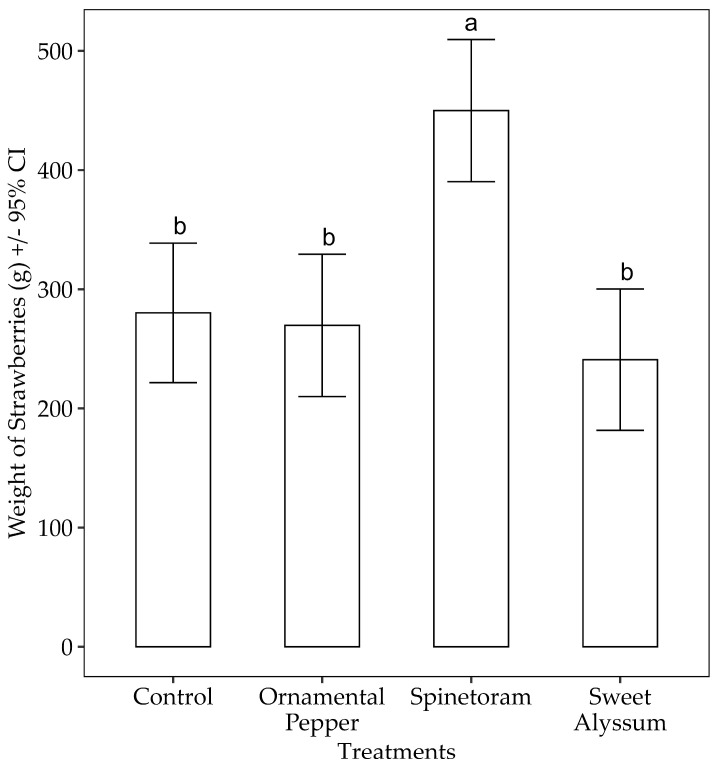
Average marketable yield of strawberries (g) per treatment during the 2022–2023 season. Marketable yield estimates (g) that show significant differences (Tukey *p* < 0.05) are indicated by different letters on the bars.

We also observed differences in the average weight of strawberries under different treatments (χ^2^ = 71.78, df = 3, *p* < 0.001); however, the interaction between the distance from the banker plants and treatments was not significant (χ^2^ = 10.00, df = 15, *p* = 0.82). Among all treatments, spinetoram treatment had the highest average marketable yield of strawberries (449 (g), CI: 390–509), while there was no significant difference between the control, ornamental pepper, and sweet alyssum treatments (Figure 8).

Furthermore, none of the treatments had a significant effect on the number of thrips predators captured on the yellow sticky cards (χ^2^ = 7.49, df = 3, *p* = 0.06). During the strawberry season, very low numbers of thrips predators were captured on sticky cards in all treatments. Similar to the 2021–2023 field study, the predominant species of thrips predators captured were *Geocoris* spp. and *Orius* spp. Sweet alyssum was found to attract higher numbers of these beneficial predators, with an average count of 0.23 (CI: 0.15–0.31), in comparison with both spinetoram, with 0.13 (CI: 0.03–0.21), and the control treatment, with 0.12 (CI: 0.02–0.211). Among all treatments, the lowest number of *Geocoris* spp. and *Orius* spp. were recorded in the ornamental pepper treatment at 0.086 (CI: 0.008–0.163).

## 4. Discussion

The overall key findings from this research indicated that the proximity of banker plants to strawberry plots could lead to a decrease in foliar damage by *S. dorsalis* particularly at 3.7 m. This correlation became more pronounced with greater distances from the banker plants as strawberry plants situated at distances exceeding 3.7 m away from the banker plants had higher foliar damage indices compared with those located in closer proximity. This discovery offers preliminary evidence regarding the optimal distance at which banker plants should be situated to achieve maximal efficacy in the field.

During the study, two thrips predators, *Geocoris* spp. and *Orius* spp., were predominantly observed within the ecosystem. Nevertheless, these predators had a low presence for the entire duration of the experiment, and this confirmed prior reports of the occurrence of these thrips predators in the study area although in relatively low numbers [44]. Furthermore, despite their presence, these thrips predators did not effectively suppress the *S. dorsalis* populations. This may be because the area covered with banker plants did not provide sufficient biomass to support enough predators to control the thrips [45,46].

In the second year (2022–2023) of the experiment, the strawberry plots treated with spinetoram had the lowest leaf damage index, the lowest occurrence of *S. dorsalis* within the strawberry plots, and the highest yield, thereby suggesting spinetoram to be the most effective treatment. Spinetoram has been used extensively in the management of various thrips species [47,48,49] and in the management of *S. dorsalis* in strawberries [5,16,18,20]. The observations performed in strawberry plots treated with spinetoram in this study aligned with those reported by Lahiri and Yambisa (2021) [18], who reported that strawberry plants treated with spinetoram had higher yields, reduced leaf damage, and lower numbers of *S. dorsalis* compared with the other treatments in their study.

In the same season, strawberry plots in close proximity to ornamental pepper had lower *S. dorsalis* populations and lower foliar damage compared with those near sweet alyssum. This observation could possibly be as a result of capsaicinoids present in the pepper plants. Capsaicinoids found in peppers have been used as a repellent for a variety of insect pests [50]. Additionally, capsaicin has been shown to be an effective oviposition deterrent for many insects [51,52]. Due to its activity, capsaicin has been commercialized as an active ingredient in several bioinsecticides. For example, Captiva^®^ Prime (Gowan, Yuma, AZ, USA), an insecticide containing Capsicum oleoresin as one of its active ingredients, has been demonstrated to effectively manage *S. dorsalis* populations in strawberries [18,50,51,52,53]. Moreover, we observed an increase in damage to plants planted farther away from the ornamental pepper, likely indicating a decrease in the concentration of the capsaicinoids in the air. Furthermore, when combined with predatory mites, particularly *A. swirskii*, ornamental peppers could contribute to the season-long suppression of *S. dorsalis* and *T. urticae* in strawberry fields. This is because ornamental peppers have been shown to support the establishment of *A. swirskii* and enhance its suppression of multiple pests in greenhouse environments [54]

Sweet alyssum attracted the highest number of thrips predators; however, these predators did not significantly reduce *S. dorsalis* numbers. This was likely because sweet alyssum also attracted other prey, such as *F. bispinosa*, which these predators could have potentially fed on, thus eliminating the need for the predators to move into strawberry beds and target *S. dorsalis*. Despite our observation, sweet alyssum as a banker plant has been recognized for its capacity to support a diverse array of beneficial insects, such as *Cotesia marginiventris* (Cresson) (Hymenoptera: Braconidae), *Diadegma insulare* (Cresson) (Hymenoptera: Ichneumonidae) [55], *Orius insidiosus* Say (Hemiptera: Anthocoridae), and various species of assassin bugs (Hemiptera: Reduviidae) [56]. Additionally, sweet alyssum was reported to attract *Orius laevigatus* for the suppression of western flower thrips [57]. The effectiveness of sweet alyssum as a banker plant is multifaceted, attributable to its prolonged flowering period that ensures a continuous supply of pollen and nectar for many beneficial insects. Moreover, it attracts fewer bees, thereby reducing competition for hoverflies, which are recognized predators of numerous insect pests [25,28].

## 5. Conclusions

Based on results from this study, it can be concluded that sweet alyssum and ornamental pepper can attract thrips predators, although in relatively low numbers that may not be sufficient to suppress *S. dorsalis* populations in strawberries but are able to decrease the foliar damage in the crop at a distance of 3.7 m from them. These findings highlight the potential of incorporating ornamental pepper into an *S. dorsalis* pest management program, not as a banker crop but for its pest-repellent properties. This scenario presents a promising avenue for future research, specifically assessing the potential of utilizing ornamental pepper as insectary habitats in the field where *Geocoris* spp. and *Orius* spp. or predatory mites could be released early in the growing season. Such an approach would facilitate the establishment of substantial populations of predatory mites, which, in turn, could effectively suppress *S. dorsalis* populations as they begin to increase. Additionally, this strategy would offer a significant advantage to growers by eliminating the need for multiple insecticide sprays, as well as releases of predatory mites.

## Data Availability

The dataset is available upon reasonable request from the authors.
